# Efficacy and Safety of Once-Weekly Trelagliptin As Compared to Twice-Daily Vildagliptin in Indian Patients With Type 2 Diabetes Mellitus: A Randomized, Phase 3, Non-inferiority Clinical Trial

**DOI:** 10.7759/cureus.85219

**Published:** 2025-06-02

**Authors:** Bhupesh Dewan, Siddheshwar Shinde, Shefali Roy

**Affiliations:** 1 Medical Services, Zuventus Healthcare Limited, Mumbai, IND

**Keywords:** dpp-4 inhibitor, efficacy and safety, once weekly, trelagliptin, types 2 diabetes

## Abstract

Background

Trelagliptin and vildagliptin are oral dipeptidyl peptidase-4 (DPP-4) inhibitors used in the treatment of type 2 diabetes mellitus. The administration of vildagliptin is twice daily, whereas trelagliptin provides the convenience of once-weekly dosing, which may enhance patient adherence. A phase 3 clinical trial was conducted to assess the non-inferiority of trelagliptin compared to vildagliptin.

Methods

This multicenter, randomized, open-label, parallel-group, active-controlled non-inferiority clinical trial was conducted at 10 geographically distinct sites across India. A total of 240 treatment-naive patients with type 2 diabetes mellitus were randomized in a 1:1 ratio to receive either trelagliptin (100 mg once weekly) or vildagliptin (50 mg twice daily) for 16 weeks. The primary endpoint was non-inferiority of trelagliptin to vildagliptin in reducing glycated hemoglobin (HbA1c) levels from baseline to week 16. Secondary efficacy measures included changes in fasting and postprandial blood glucose, fasting insulin, glucagon, C-peptide, and glucagon-like peptide-1 (GLP-1) levels. Safety was assessed based on the incidence of adverse events.

Results

At week 16, the mean HbA1c levels were 7.18 ± 1.47% and 7.21 ± 1.49% in trelagliptin and vildagliptin groups, respectively (Δ -0.89% vs. Δ -1.00%, p < 0.0001). The difference between groups was 0.11% (95% CI: -0.28 to 0.50; p = 0.5899), showing non-inferiority of trelagliptin. A total of 48.57% of patients in the trelagliptin group and 47.57% in the vildagliptin group achieved the target HbA1c level of <7% (p = 0.8850). No statistically significant differences were observed between the groups for glycemic parameters, including fasting blood glucose (Δ 1.11; 95% CI: -16.79 to 19.02; p = 0.9025), 2-hr postprandial glucose (Δ 3.33; 95% CI: -30.55 to 23.88; p = 0.8093), fasting serum insulin (Δ 5.22; 95% CI: -15.01 to 25.45; p = 0.6113), fasting glucagon (Δ 0.72; 95% CI: -96.34 to 94.90; p = 0.9882), C-peptide (Δ 0.36; 95% CI: -0.31 to 1.03; p = 0.2912), and GLP-1 levels (Δ -0.02; 95% CI: -0.06 to 0.02; p = 0.3995). All reported adverse events were mild in nature and resolved without any lasting effects. Adverse events occurred in 6.67% (8/120) of patients in the trelagliptin group and 9.17% (11/120) in the vildagliptin group.

Conclusions

Trelagliptin showed a significant reduction in HbA1c, fasting, and postprandial glucose levels, indicating effective glycemic control in patients with type 2 diabetes mellitus. The study drug exhibited a favorable safety profile, with no major adverse events reported. Overall, trelagliptin proved to be both efficacious and well-tolerated, demonstrating non-inferiority to vildagliptin.

## Introduction

Diabetes poses a major global public health challenge, affecting both health outcomes and socioeconomic progress. The International Diabetes Federation projects that by 2050, diabetes will affect around 853 million people globally, representing a 46% increase from present numbers [1]. According to the 2023 Indian Council of Medical Research - India Diabetes (ICMR INDIAB) study, the prevalence of diabetes in India stands at 101 million [2]. The relative increase in prevalence was approximately 40% in 25 years [3] and is expected to be 125 million in the next 25 years [4]. According to the National Family Health Survey (NFHS-5), 16.1% of adults aged 15 and older have diabetes [5].

Despite the availability of effective anti-diabetics, only 57.3% of patients reach the target glycated hemoglobin (HbA1c) level of less than 7.0% [6]. Patient adherence in diabetes management is crucial for achieving optimal glycemic control and preventing complications. However, adherence to daily medication regimens can be challenging due to factors such as long duration of treatment, the complexity of treatment regimens, frequent dosing schedules, and the need to take multiple medications [7]. A 10% increase in non-adherence to diabetic treatment is associated with an increase of 0.14% in HbA1c [8, 9].

A daily dosing regimen can support glycemic control, but it often requires more effort to maintain adherence, which can increase the burden on patients. By reducing the dosing frequency and extending the drug’s duration of action, treatment becomes simpler, promoting better adherence [10]. Transitioning to a once-weekly dipeptidyl peptidase (DPP)-4 inhibitor has been shown to be both effective and well tolerated in diabetes management, resulting in improved patient compliance, satisfaction, and quality of life. It also eases the burden on caregivers, particularly for older individuals [11].

Once-weekly trelagliptin is a novel, orally active, highly selective DPP-4 inhibitor that has been approved for use in type 2 diabetes mellitus in Japan [12]. DPP-4 inhibitors promote insulin secretion in a blood glucose-dependent manner, reducing the risk of hypoglycemia. Trelagliptin effectively reduces glycemic variability and improves glycemic control without causing hypoglycemia [13,14]. Trelagliptin is weight-neutral like other DPP-4 inhibitors and generally well-tolerated, making them appropriate for a wide variety of patients [14].

Trelagliptin is a selective and potent competitive inhibitor of DPP-4, known for its long-lasting effects. Trelagliptin, a fluorinated derivative of alogliptin, exhibits enhanced potency approximately 4-fold greater than alogliptin and 12-fold greater than sitagliptin, attributed to the strategic fluorine modification [15]. The fluorine substitution in trelagliptin slows its dissociation rate by approximately 8-fold than alogliptin (29 min vs. 3.7 min). Trelagliptin demonstrated IC50 >100,000 nmol/L corresponding to over 10,000-fold selectivity for DPP-4 enzyme as compared to related proteases DPP-2, DPP-8, DPP-9, PEP, and FAPα [15]. Considering the pharmacological and clinical benefits, a phase 3 clinical trial in Indian patients with type 2 diabetes mellitus was conducted to evaluate the efficacy and safety of once-weekly trelagliptin compared to vildagliptin, an approved and commonly prescribed medication in India.

## Materials and methods

Study design and ethics

This study was a multicentric, randomized, open-label, parallel, comparative, active-controlled phase 3 clinical trial in the management of type 2 diabetes mellitus. The study was conducted according to New Drugs and Clinical Trials, Rules, 2019, Ethical Guidelines for Biomedical Research on Human Participants, Indian Council of Medical Research, 2017, International Council for Harmonization Guidelines (ICH) E6 (R2) for Good Clinical Practice, Declaration of Helsinki (Brazil, October 2013). The clinical trial was registered prospectively with the Clinical Trial Registry of India (Reg. No: CTRI/2023/01/048826 dated 9 January 2023).

Study population

The study was conducted between 09 February 2023 and 04 April 2024 across 10 hospitals located throughout India which included: Kurnool Medical College and Government General Hospital (Kurnool), Pranaam Hospital (Hyderabad), Karnataka Institute of Medical Sciences (Hubli), Grant Government Medical College and Sir J.J. Group of Hospitals (Mumbai), 7-Orange Hospital (Pune), Shree Siddhivinayak Hospital (Nashik), Max Super Specialty Hospital (Ghaziabad), Maya Hospital (Kanpur), Rajendra Institute of Medical Sciences (Ranchi), and Roy Hospital (Siliguri). The participants were enrolled based on predefined inclusion and exclusion criteria following a screening procedure. Eligible participants were adults (≥18 years) with a body mass index (BMI) of 19-35 kg/m2, newly diagnosed with type 2 diabetes mellitus with HbA1c levels ranging from 6.5 to 10% (both inclusive). Exclusion criteria included history of acute or chronic liver disease, elevated liver enzymes (aspartate aminotransferase (AST) or alanine aminotransferase (ALT) >2.5 times of reference range), total bilirubin >1.5 times of reference range, renal impairment (estimated glomerular filtration rate (eGFR) <60 mL/min/1.73 m2 or serum creatinine >1.5 times of reference range), history of pancreatitis, significant cardiovascular conditions (including myocardial infarction, unstable angina, or heart failure), neurological or psychiatric disorders, untreated thyroid abnormalities, organ transplantation, severe pulmonary disease, and other serious systemic illnesses. Pregnant or lactating women, and those not using adequate contraception, were also excluded.

Study treatment

**Figure 1:**
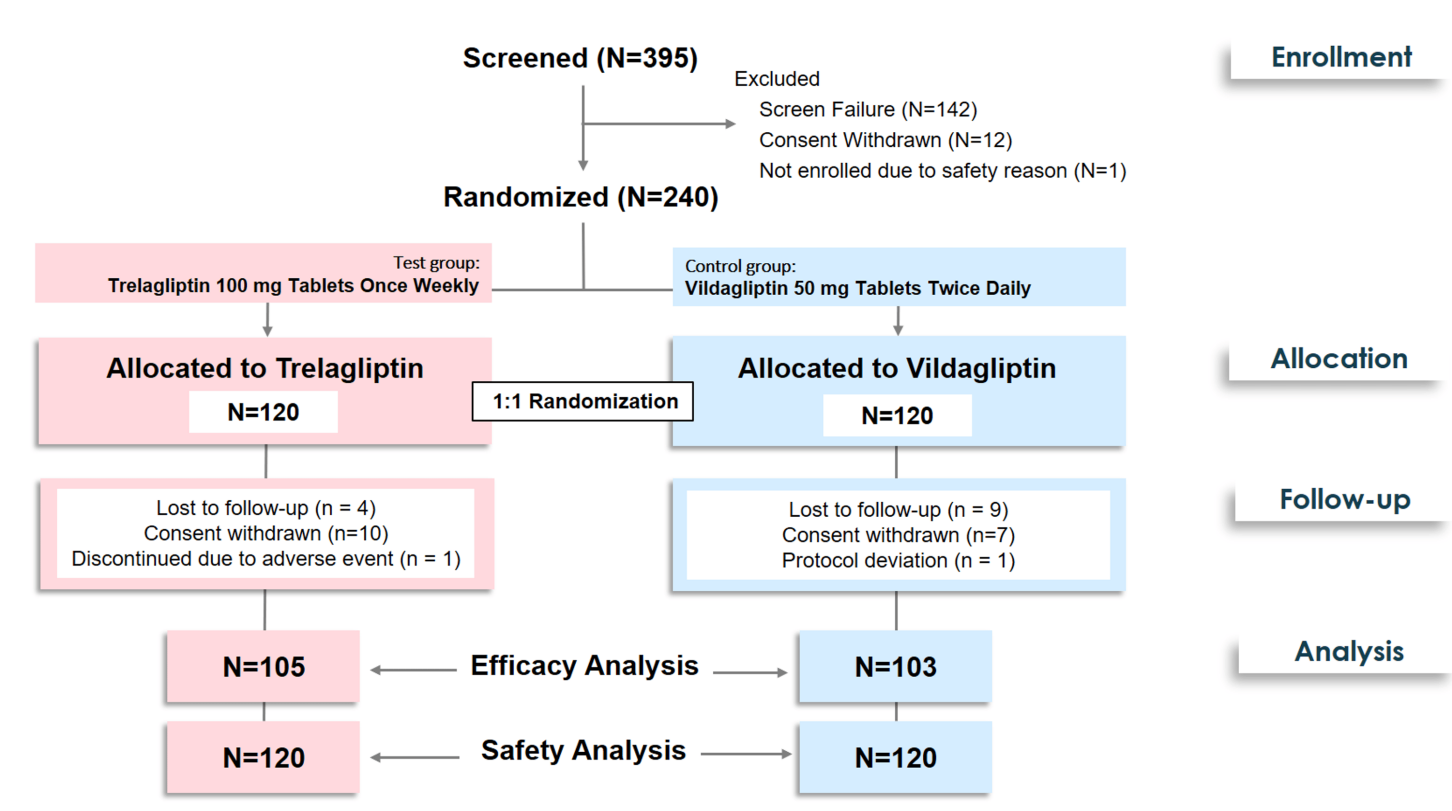
CONSORT flowchart for the disposition of patients in the study

Eligible patients were randomly assigned to one of two treatment groups using a computer-generated block randomization method using SAS software version 9.4 (SAS Institute Inc., USA) [16]. The randomization ensured balanced allocation in a 1:1 ratio to either the trelagliptin group or the vildagliptin group. Patients in the test group received trelagliptin 100 mg tablets (Zuventus Healthcare Limited) once weekly, while those in the control group received vildagliptin 50 mg tablets (Zuventus Healthcare Limited) twice daily for a duration of 16 weeks. Participants were instructed to take their assigned study medication at the same time each day or week, depending on the dosing schedule. The subject disposition is illustrated in Figure 1.

Study procedure

All eligible participants confirmed their willingness to participate by signing the ethics committee-approved informed consent form prior to enrollment. During the informed consent process, patients were informed about the study procedures, the investigational products, and potential risks and benefits. After the screening period, we randomly assigned eligible patients in a 1:1 ratio to receive 16 weeks of treatment with either trelagliptin or vildagliptin. Based on the schedule, the study drugs were then dispensed to the participants. Patients were monitored at 6- and 12-week post-randomization to assess fasting blood glucose and 2-hour postprandial blood glucose levels, and to evaluate any adverse events. At the end of the treatment period (week 16), patients attended a final study visit, during which their glycemic control was evaluated. Vital signs and key laboratory parameters were also assessed. Treatment compliance was evaluated through a review of the patients’ diaries. All patients were given a diary to record dosing details of the assigned treatment. At each follow-up, the principal or co-investigator assessed treatment compliance by verifying used, unused, lost, or damaged study drug units against the patient diary entries.

Study assessment

The primary endpoint was non-inferiority of trelagliptin as compared with vildagliptin in terms of glycemic control, measured by the difference in mean HbA1c levels from baseline to week 16. Secondary endpoints included changes in fasting blood glucose, 2-hour postprandial blood glucose, fasting insulin, fasting glucagon, C-peptide, glucagon-like peptide-1 (GLP-1), and body weight from baseline to week 16. Safety was evaluated through the incidence of adverse events, vital signs, and laboratory parameters, including complete blood count, complete urine analysis, liver function test, and renal function tests.

Statistical analysis

The mean difference in HbA1c reduction between the trelagliptin and alogliptin treatment groups was 0.11% ± 0.86 [17]. To show non-inferiority of trelagliptin compared with vildagliptin, between-group differences in HbA1c were evaluated with a non-inferiority limit of 5%. The sample size was calculated assuming a mean HbA1c reduction of 0.14% between groups with a standard deviation of 0.20. Based on a 5% significance level and 90% statistical power, a sample size of 104 patients per treatment group was required. Considering a 10% drop-out rate, 240 patients (120 per group) were enrolled in this study.

Descriptive statistics of demographics and other baseline characteristics were presented for all patients for both groups. Descriptive statistics will be used to summarize baseline characteristics. The baseline characteristics were assessed using an unpaired t-test. Change from baseline to week 16 was analyzed using a paired t-test in both groups. Differences in change from baseline between treatment groups were compared using the unpaired t-test. The values are presented in means ± SD with a 95% CI unless otherwise indicated. A 5% level of significance was considered for statistical analysis. Safety was analyzed based on the number of adverse events observed and the total number of patients reporting adverse events. The proportion of patients experiencing adverse events was compared between the two treatment groups using the chi-square or Fisher’s exact test. All statistical analysis was performed using the SAS software version 9.4 (SAS Institute Inc., USA) [16].

## Results

A total of 395 patients were screened. Among these, 240 patients who met the eligibility criteria were randomized and treated with trelagliptin (n=120) and vildagliptin (n=120) as per the randomization schedule. The 16-week treatment period was completed by 208 patients and considered for the efficacy analysis (Figure 1). Patients who had received at least one dose of the assigned treatment were included in the safety analysis.

The mean (±SD) age of the subjects was 47.17 (±11.82) years in the test group and 44.59 (±10.94) years in the control group. The mean (±SD) of BMI was 26.35 (±3.23) kg/m^2^ in the test group and 26.34 (±3.34) kg/m^2^ in the control group. Demographic and clinical characteristics were well distributed between the groups, and no significant differences between groups were observed (Table 1).

**Table 1:** Baseline characteristics ALT: alanine aminotransferase; AST: aspartate aminotransferase; BMI: body mass index; BUN: blood urea nitrogen; DBP: diastolic blood pressure; eGFR: estimated glomerular filtration rate; GLP-1: glucagon-like peptide-1; HbA1c: glycated hemoglobin; SBP: systolic blood pressure Values are presented as mean ± SD, except for gender, which is the number of patients (%). * t-value (test statistic) from the unpaired t-test. ** chi-square value (test statistic) from the Pearson chi-square test. ^#^Data analyzed using the unpaired t-test. ^##^Data analyzed using the Pearson chi-square test.

Baseline Characteristics	Trelagliptin (N=120)	Vildagliptin (N=120)	Test Statistics*	p-value^#^
Age (years)	47.17 ± 11.82	44.59 ± 10.94	1.7519	0.0811
Gender				
Male	74 (61.67)	82 (68.33)	1.1722**	0.2790^##^
Female	46 (38.33)	38 (31.67)		
Body weight (kg)	69.45 ± 9.47	69.89 ± 10.18	-0.3479	0.7282
BMI (kg/m^2^)	26.35 ± 3.23	26.34 ± 3.34	0.0177	0.9859
HbA1c (%)	8.02 ± 1.07	8.25 ± 1.15	-1.6005	0.1108
Fasting plasma glucose (mg/dL)	153.07 ± 49.72	157.14 ± 60.88	-0.5679	0.5706
2-hr postprandial glucose (mg/dL)	232.33 ± 70.69	241.42 ± 99.56	-0.8149	0.4160
Fasting insulin (IU/mL)	14.56 ± 13.35	17.11 ± 35.71	-0.7313	0.4653
Fasting glucagon (pg/mL)	187.90 ± 160.50	204.16 ± 188.65	-0.7192	0.4727
C-peptide (ng/mL)	3.10 ± 1.76	3.17 ± 2.57	-0.2474	0.8048
GLP-1 (pmol/L)	0.09 ± 0.17	0.08 ± 0.09	0.8054	0.4214
SBP (mmHg)	124.07 ± 7.55	125.11 ± 9.24	-0.9475	0.3444
DBP (mmHg)	80.77 ± 5.36	81.69 ± 5.51	-1.3188	0.1885
Pulse rate (beats/min)	83.08 ± 8.19	82.79 ± 8.20	0.2758	0.7830
Serum creatinine (mg/dL)	0.76 ± 0.22	0.77 ± 0.22	-0.3528	0.7245
eGFR (mL/min/1.73 m^2^)	105.75 ± 17.80	107.93 ± 18.73	-0.9256	0.3556
BUN (mg/dL)	11.06 ± 4.62	11.14 ± 3.78	-0.1499	0.8810
AST (U/L)	32.80 ± 15.00	32.35 ± 15.21	0.2303	0.8180
ALT (U/L)	35.47 ± 21.17	34.30 ± 17.98	0.4608	0.6454
Total bilirubin (mg/dL)	0.57 ± 0.34	0.58 ± 0.32	-0.1170	0.9069
Amylase (U/L)	77.83 ± 34.13	75.25 ± 33.94	0.5880	0.5571
Lipase (U/L)	41.83 ± 32.57	43.19 ± 27.14	-0.3512	0.7258

The mean change in HbA1c from baseline to the end of treatment (week 16) was -0.89% with trelagliptin and -1.00 % with vildagliptin, with a mean difference of 0.11% (95% CI: -0.28 to 0.50; p=0.5899). The upper limit of the 95% CI was below the predefined non-inferiority margin of 5%; therefore, trelagliptin 100 mg once a week was non-inferior to vildagliptin 50 mg twice daily. The response rate, defined as the proportion of patients achieving HbA1c <7.0% at treatment end, was similar between the trelagliptin (48.57%) and vildagliptin (47.57%) groups, with no significant difference (p = 0.8850).

**Figure 2:**
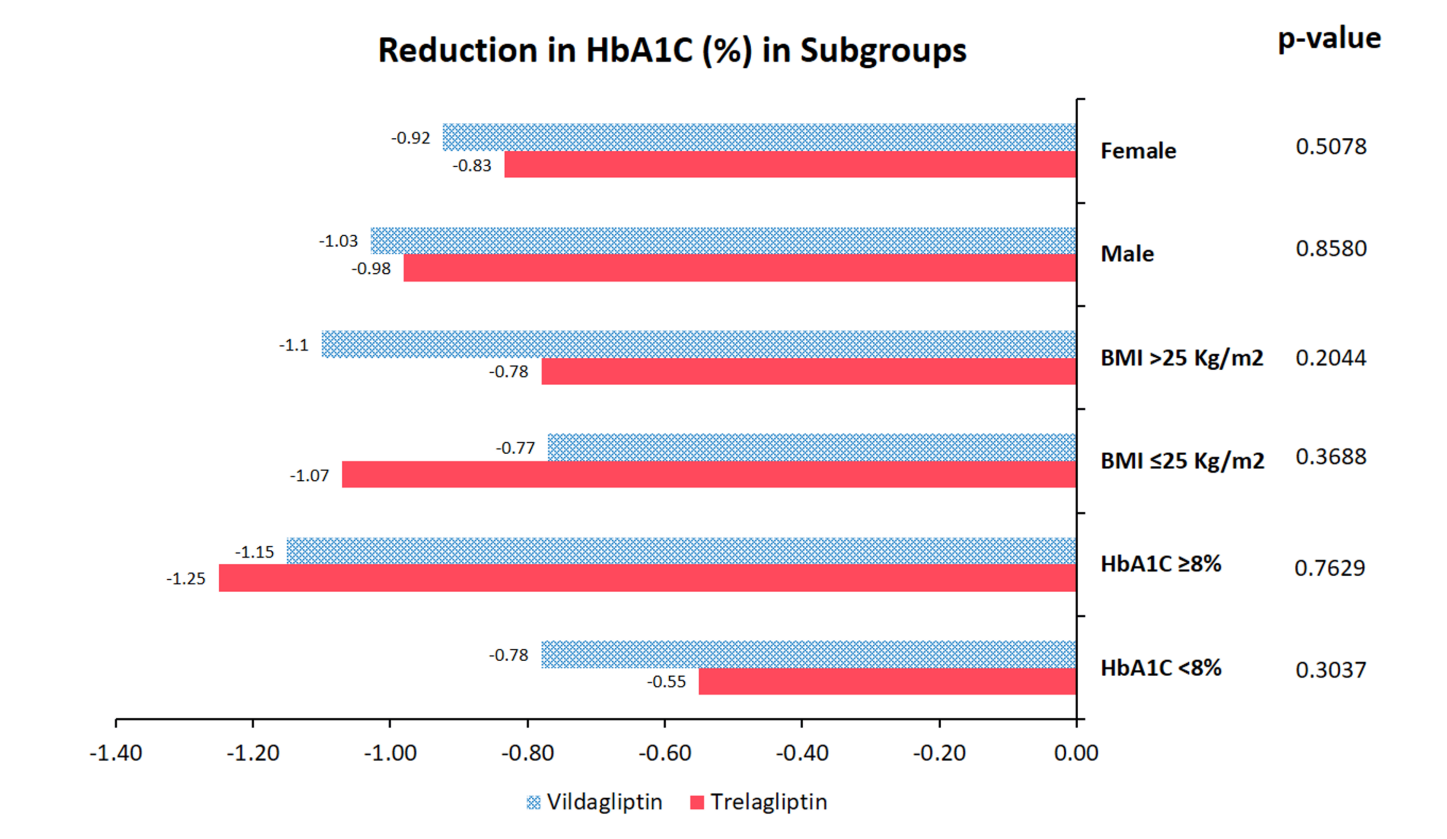
Subgroup analysis Mean changes of HbA1c (%) from baseline to the end of treatment (16 weeks) in patient subgroups defined by gender, BMI (≤25 or >25 kg/m^2^), and baseline HbA1c values (<8 or ≥8 %). The p-values (calculated using an unpaired t-test) indicate there are no statistically significant differences in the reduction of HbA1c across the respective subgroups when comparing once-weekly trelagliptin with twice-daily vildagliptin.

Patients were stratified by gender, BMI (≤25 or >25 kg/m^2^), and baseline HbA1c (<8% or ≥8%). Overall, the mean changes in HbA1c from baseline to week 16 were lower across all subgroups, and no statistically significant differences in HbA1c reduction were observed between the groups. Figure 2 illustrates the changes in HbA1c levels from baseline within each subgroup.

The mean change in fasting blood glucose (in mg/dL) from baseline to week 16 was -14.89 (p=0.0181) with trelagliptin and -16.00 (p=0.0179) with vildagliptin, giving a mean difference of 1.11 (95% CI: -16.79 to 19.02; p=0.9025). Reduction in 2-hr postprandial plasma glucose (in mg/dL) after 16 weeks of treatment was significantly reduced from baseline in both groups (Trelagliptin group, -48.20, p<0.0001; vildagliptin group, -44.86, p<0.0001). The change between the groups did not differ significantly. The trelagliptin and vildagliptin groups showed no notable differences in terms of insulin, fasting glucagon, C-peptide, and GLP-1 levels (Table 2). Change in body weight (in kg) was not significant in trelagliptin (69.45 ± 9.47 to 68.87 8.76; p=0.6337) and in vildagliptin (69.89 ± 10.18 to 70.05 ± 9.82; p=0.9077) groups after 16 weeks of treatment.

**Table 2:** Change in efficacy parameters from baseline to the end of the treatment period GLP-1: glucagon-like peptide-1; HbA1c: glycated hemoglobin *Baseline and week 16 data for trelagliptin (N=105) and vildagliptin (N=103) are presented in mean ± SD. **Mean difference and 95% CI represent within-group changes from baseline to week 16, analyzed using a paired t-test. ^##^ Mean difference and 95% CI represent between-group comparisons at week 16, analyzed using an unpaired t-test.

	Baseline*	Week 16*	Within group		Between group	
			Mean difference (95% CI)**	p-value**	Mean difference (95% CI)^##^	p-value^##^
HbA1c (%)						
Trelagliptin	8.07 ± 1.08	7.18 ± 1.47	-0.89 (-1.17 to -0.61)	<0.0001	0.11 (-0.28 to 0.50)	0.5899
Vildagliptin	8.21 ± 1.14	7.21 ± 1.49	-1.00 (-1.27 to -0.72)	<0.0001		
Fasting blood glucose (mg/dL)						
Trelagliptin	150.97 ± 47.66	136.09 ± 44.51	-14.89 (-27.18 to -2.60)	0.0181	1.11 (-16.79 to 19.02)	0.9025
Vildagliptin	157.07 ± 62.20	141.07 ± 55.04	-16.00 (-29.18 to -2.82)	0.0179		
2-hr postprandial glucose (mg/dL)						
Trelagliptin	232.01 ± 70.35	183.81 ± 67.93	-48.20 (-66.05 to -30.35	<0.0001	3.33 (-30.55 to 23.88)	0.8093
Vildagliptin	238.59 ± 99.45	193.73 ± 74.20	-44.86 (-65.68 to -24.05	<0.0001		
Fasting insulin (IU/mL)						
Trelagliptin	13.25 ± 11.58	26.62 ± 72.39	13.36 (-0.72 to 27.45)	0.0627	5.22 (-15.01 to 25.45)	0.6113
Vildagliptin	17.78 ± 38.30	25.92 ± 92.36	8.14 (-6.55 to 22.83)	0.2742		
Fasting glucagon (pg/mL)						
Trelagliptin	191.96 ± 165.64	405.51 ± 311.92	213.55 (145.07 to 282.04)	<0.0001	0.72 (-96.34 to 94.90)	0.9882
Vildagliptin	186.71 ± 161.93	400.98 ± 337.23	214.27 (146.76 to 281.78)	<0.0001		
C-peptide (ng/mL)						
Trelagliptin	2.93 ± 1.55	2.87 ± 1.90	-0.06 (-0.40 to 0.29)	0.7474	0.36 (-0.31 to 1.03)	0.2912
Vildagliptin	3.23 ± 2.74	2.81 ± 1.91	-0.35 (-1.00 to 0.17)	0.1605		
GLP-1 (pmol/L)						
Trelagliptin	0.10 ± 0.18	0.16 ± 0.12	0.06 (0.02 to 0.10)	0.0030	-0.02 (-0.06; 0.02)	0.3995
Vildagliptin	0.09 ± 0.09	0.17 ± 0.12	0.08 (0.05 to 0.11)	<0.0001		

All adverse events were of mild severity and resolved without any sequelae at the end of the study. A total of 6.67% of subjects experienced adverse events in the test group, whereas 9.17% in the control group. It was observed that there was a statistically non-significant difference (p=0.4730) between the two treatments in the incidence of adverse events. Both treatments were well-tolerated by the patients. Pancreatitis was not observed in any enrolled patients. Hypoglycemia was not reported in the trelagliptin group, whereas one incidence was observed in the vildagliptin group. No cases of any event related to cardiovascular disease, death, or serious adverse event leading to discontinuation of the study drug were reported. No clinically relevant differences in laboratory tests were noted between the groups (Table 3). Treatment compliance was assessed at each visit by recording it in the patient diary. Overall compliance was excellent, with all patients identified as taking trelagliptin, with dosage compliance being 99% vs. 96% for vildagliptin.

**Table 3:** Safety evaluation based on the changes in laboratory parameters ALT: Alanine aminotransferase; AST: aspartate aminotransferase; BUN: blood urea nitrogen; eGFR: estimated glomerular filtration rate * Baseline (Trelagliptin N=120 and vildagliptin N=120) and week 16 (Trelaglipin N=105 and vildagliptin (N=104) data are presented in mean ± SD. ** Mean difference and 95% CI represent within-group changes from baseline to week 16, analyzed using a paired t-test. ^##^Mean difference and 95% CI represent between-group comparisons at week 16, analyzed using an unpaired t-test.

Lab Investigations	Trelagliptin				Vildagliptin				Mean Diff. between groups (95% CI)^##^	p-value^##^
	Baseline*	Week 16*	Mean Diff (95%CI)**	p-value**	Baseline*	Week 16*	Mean Diff (95%CI)**	p-value**		
Pancreatic Function Tests										
Amylase (U/L)	77.83 ± 34.13	79.47 ± 35.15	0.31 (-6.20; 6.83)	0.9240	75.25 ± 33.94	69.68 ± 25.96	-5.33 (-12.64; 1.99)	0.1520	5.64 (-4.10; 15.38)	0.2548
Lipase (U/L)	41.83 ± 32.57	45.80 ± 37.61	4.17 (-4.40; 12.73)	0.3368	43.19 ± 27.14	41.26 ± 27.63	-1.11 (-7.38; 5.17)	0.7275	5.27 (-5.30; 15.84)	0.3266
Renal Function Tests										
Serum creatinine (mg/dL)	0.76 ± 0.22	0.75 ± 0.18	-0.02 (0.07; 0.03)	0.3779	0.77 ± 0.22	0.78 ± 0.21	0.01 (-0.04; 0.04)	0.9990	-0.02 (-0.08; 0.04)	0.5171
eGFR (mL/min/1.73m^2^)	105.75 ± 17.80	106.74 ± 16.72	1.04 (-2.82; 4.90)	0.5948	107.93 ± 18.73	107.07 ± 17.20	-0.20 (-3.88; 3.48)	0.9135	1.24 (-4.06; 6.54)	0.6452
BUN (mg/dL)	11.06 ± 4.62	11.28 ± 3.79	0.04 (-0.89; 0.97)	0.9323	11.14 ± 3.78	11.21 ± 3.58	-0.08 (-0.99; 0.82)	0.8563	0.12 (-1.17; 1.41)	0.8514
Liver Function Tests										
AST (U/L)	32.80 ± 15.00	38.11 ± 25.34	4.88 (0.02; 9.73)	0.0491	32.35 ± 15.21	37.66 ± 25.39	5.56 (0.21; 10.91)	0.0418	-0.69 (-7.87; 6.49)	0.8505
ALT (U/L)	35.47 ± 21.17	36.83 ± 21.21	0.78 (-3.72; 5.29)	0.7303	34.30 ± 17.98	38.49 ± 33.07	4.30 (-2.37; 10.96)	0.2037	-3.51 (-11.49; 4.47)	0.3865
Total bilirubin (mg/dL)	0.57 ± 0.34	0.64 ± 0.42	0.07 (-0.03; 0.17)	0.1595	0.58 ± 0.32	0.64 ± 0.32	0.10 (0.03; 0.16)	0.0051	-0.02 (-0.14; 0.09)	0.6915

## Discussion

Adherence to oral antidiabetic medications varies widely, with rates ranging from 33% to 93% [18]. Notably, around two-thirds of individuals with diabetes face challenges in maintaining adequate adherence to their prescribed treatment. Various factors contribute to this challenge, including the complexity of treatment regimens, which may involve multiple drugs or daily doses, as well as issues like difficulties in remembering to take medications or refill prescriptions. Additionally, more than half of patients on daily antidiabetic therapy expect a reduction in the number and types of medications they need to take [19-22]. Patients with chronic conditions often prefer less frequent dosing schedules, finding them more convenient than daily regimens. As a result, a once-weekly oral antidiabetic medication may enhance adherence by reducing the burden of daily intake [23,24]. Patients receiving weekly DPP-4 inhibitors reported significantly greater satisfaction with convenience and flexibility compared to those on daily DPP-4 inhibitors [10].

In a phase 2 Japanese trial, trelagliptin demonstrated a dose-dependent reduction in HbA1c at doses ranging from 12.5 to 200 mg, which were statistically significant compared to the placebo. At 100 mg dosing, the mean rate of DPP-4 inhibition remained approximately 75-80% at 7 days after dosing [25]. In a phase 3 Japanese trial, trelagliptin showed comparable efficacy and safety to alogliptin 25 mg, with the inhibition of DPP-4 in the trelagliptin 100 mg group showing no significant differences from the alogliptin group [17]. Additionally, in a phase 3 long-term study, trelagliptin exhibited good long-term safety and efficacy [26]. These findings reinforce the potential of trelagliptin, which is suitable for once-weekly administration. With its convenient dosing schedule, trelagliptin is expected to improve patient adherence and help prevent complications.

In this randomized trial, naïve type 2 diabetes mellitus patients received either trelagliptin once weekly or vildagliptin twice daily for 16 weeks. The primary outcome was the mean change in HbA1c from baseline to week 16, which exhibited significant improvements in HbA1c (p<0.0001). Furthermore, both treatments led to similar reductions in blood glucose levels (both fasting and postprandial) and showed comparable changes in fasting insulin, fasting glucagon, and GLP-1 levels. This novel DPP-4 inhibitor effectively controlled glycemic parameters without any severe adverse events during the 16-week treatment period.

The present study showed that once-weekly trelagliptin was non-inferior in efficacy to the twice-daily DPP-4 inhibitor, vildagliptin. Trelagliptin was also well tolerated, and no significant adverse events were reported during the study. Findings from a 52-week study support the long-term efficacy of trelagliptin as both monotherapy and in combination therapy [26]. These results suggest that trelagliptin may be a suitable alternative and can be considered as a switch from daily DPP-4 inhibitors without compromising glycemic control and safety [11].

This study had some limitations. Its open-label design may introduce potential biases. Furthermore, as the study focused solely on treatment-naive patients with diabetes, data on the switchover from existing antidiabetic therapies were not available. There remains a paucity of data on renal impairment among Indian patients. Additionally, this study involved a short follow-up period, highlighting the need for long-term studies. Therefore, further randomized controlled trials and post-marketing surveillance are required to address these limitations and validate the findings.

## Conclusions

In summary, a 16-week treatment with trelagliptin (100 mg once weekly) significantly improved glycemic control, as primarily evidenced by reductions in HbA1c, fasting, and postprandial glucose levels in drug-naïve patients with type 2 diabetes mellitus. These efficacy outcomes were comparable to those observed with vildagliptin (50 mg twice daily) treatment. Both treatments were well tolerated. Given its non-inferior glucose-lowering efficacy and favorable safety profile compared to vildagliptin, trelagliptin represents a convenient and effective once-weekly therapeutic option for the management of type 2 diabetes mellitus.

## References

[1] (2025). International diabetes federation. IDF Diabetes Atlas 11th edition. https://idf.org/about-diabetes/diabetes-facts-figures/.

[2] Anjana RM, Unnikrishnan R, Deepa M (2023). ICMR-INDIAB Collaborative Study Group: Metabolic non-communicable disease health report of India: The ICMR-INDIAB national cross-sectional study (ICMR-INDIAB-17). Lancet Diabetes Endocrinol.

[3] Tandon N, Anjana RM, Mohan V (2018). The increasing burden of diabetes and variations among the states of India: The Global Burden of Disease Study 1990-2016. Lancet Glob Health.

[4] (2025). International Diabetes Federation. IDF Diabetes Atlas. 10th edition. https://www.ncbi.nlm.nih.gov/books/NBK581934/.

[5] Maiti S, Akhtar S, Upadhyay AK, Mohanty SK (2023). Socioeconomic inequality in awareness, treatment and control of diabetes among adults in India: Evidence from National Family Health Survey of India (NFHS), 2019-2021. Sci Rep.

[6] Kaku K (2015). First novel once-weekly DPP-4 inhibitor, trelagliptin, for the treatment of type 2 diabetes mellitus. Expert Opin Pharmacother.

[7] Johnston SS, Nguyen H, Felber E, Cappell K, Nelson JK, Chu BC, Kalsekar I (2014). Retrospective study of adherence to glucagon-like peptide-1 receptor agonist therapy in patients with type 2 diabetes mellitus in the United States. Adv Ther.

[8] Pladevall M, Williams LK, Potts LA, Divine G, Xi H, Lafata JE (2004). Clinical outcomes and adherence to medications measured by claims data in patients with diabetes. Diabetes Care.

[9] García-Pérez LE, Alvarez M, Dilla T, Gil-Guillén V, Orozco-Beltrán D (2013). Adherence to therapies in patients with type 2 diabetes. Diabetes Ther.

[10] Tosaki T, Kamiya H, Yamamoto Y (2017). Efficacy and patient satisfaction of the weekly DPP-4 inhibitors Trelagliptin and omarigliptin in 80 Japanese patients with type 2 diabetes. Intern Med.

[11] Oita M, Miyoshi H, Ono K (2018). Satisfaction and efficacy of switching from daily dipeptidyl peptidase-4 inhibitors to weekly trelagliptin in patients with type 2 diabetes-Randomized controlled study. Endocr J.

[12] McKeage K (2015). Trelagliptin: First Global Approval. Drugs.

[13] Nishimura R, Osonoi T, Koike Y, Miyata K, Shimasaki Y (2019). A randomized pilot study of the effect of Trelagliptin and Alogliptin on glycemic variability in patients with type 2 diabetes. Adv Ther.

[14] Dutta D, Mohindra R, Surana V, Sharma M (2022). Safety and efficacy of once weekly dipeptidyl-peptidase-4 inhibitor trelagliptin in type-2 diabetes: A meta-analysis. Diabetes Metab Syndr.

[15] Grimshaw CE, Jennings A, Kamran R (2016). Trelagliptin (SYR-472, Zafatek), novel once-weekly treatment for type 2 diabetes, inhibits dipeptidyl peptidase-4 (DPP-4) via a non-covalent mechanism. PLoS One.

[16] (2024). SAS Enterprise Guide. SAS software, version 9.4. SAS Enterprise Guide, SAS software (version 9.4). SAS Institute Inc.

[17] Inagaki N, Onouchi H, Maezawa H (2015). Once-weekly trelagliptin versus daily alogliptin in Japanese patients with type 2 diabetes: A randomised, double-blind, phase 3, non-inferiority study. The lancet Diabetes & endocrinol.

[18] Ekenberg M, Qvarnström M, Sundström A, Martinell M, Wettermark B (2024). Socioeconomic factors associated with poor medication adherence in patients with type 2 diabetes. Eur J Clin Pharmacol.

[19] Odegard PS, Capoccia K (2007). Medication taking and diabetes: A systematic review of the literature. Diabetes Educ.

[20] Bartels D (2004). Adherence to oral therapy for type 2 diabetes: Opportunities for enhancing glycemic control. J Am Acad Nurse Pract.

[21] Jabbour S, Ziring B (2011). Advantages of extended-release metformin in patients with type 2 diabetes mellitus. Postgrad Med.

[22] Wang L, Sun X, Du L, Yuan Q, Li H, Tian H, Li Y (2011). Effects and patient compliance of sustained-release versus immediate-release glipizides in patients with type 2 diabetes mellitus: a systematic review and meta-analysis. J Evid Based Med.

[23] Kruk ME, Schwalbe N (2006). The relation between intermittent dosing and adherence: Preliminary insights. Clin Ther.

[24] Recker RR, Gallagher R, MacCosbe PE (2005). Effect of dosing frequency on bisphosphonate medication adherence in a large longitudinal cohort of women. Mayo Clin Proc.

[25] Inagaki N, Onouchi H, Sano H, Funao N, Kuroda S, Kaku K (2014). SYR-472, a novel once-weekly dipeptidyl peptidase-4 (DPP-4) inhibitor, in type 2 diabetes mellitus: A phase 2, randomised, double-blind, placebo-controlled trial. Lancet Diabetes Endocrinol.

[26] Inagaki N, Sano H, Seki Y, Kuroda S, Kaku K (2016). Long-term safety and efficacy of a novel once-weekly oral trelagliptin as monotherapy or in combination with an existing oral antidiabetic drug in patients with type 2 diabetes mellitus: A 52-week open-label, phase 3 study. J Diabetes Investig.

